# Successful surgical treatment of a giant right ventricular myxoma with pulmonary artery tumor thrombus in an adolescent patient: case report

**DOI:** 10.3389/fcvm.2026.1800850

**Published:** 2026-05-11

**Authors:** Min Zeng, Bishwas Gurung, Qiang Guo, Li-Qiang Xu, Nan Hu, Jun Zhang, Xiang-Yu Luo, Qiang Liu

**Affiliations:** 1 Department of Cardiothoracic Surgery, Taihe Hospital, Hubei University of Medicine, Shiyan, China; 2 Department of General Surgery, Pushpanjali Hospital Private Limited, Chitwan, Bagmati Province, Nepal

**Keywords:** Cushing's syndrome, CT, pulmonary embolism, right ventricular myxoma

## Abstract

Right ventricular myxoma (RVM) is a rare cardiac tumor. We report an uncommon case of RVM without Cushing's syndrome in an 18-year-old male who presented with chest tightness. Echocardiography revealed an abnormal echo-dense mass (7 cm × 6.5 cm × 4.9 cm) in the right ventricle, which was strongly suggestive of myxoma. Enhanced chest computed tomography (CT) demonstrated an irregularly enhancing mass (approximately 4.7 cm × 4.3 cm) attached to the ventricular wall and septum. A nodular filling defect (approximately 2.3 cm × 1.6 cm) in the right lower pulmonary artery trunk indicated a tumor-related embolism. Laboratory tests showed normal adrenocorticotropic hormone levels, with no clinical signs of Cushing's syndrome. On the second day after admission, the patient underwent successful surgical resection of the RVM, pulmonary artery myxoma thrombus, and tricuspid valvuloplasty. Postoperative recovery was uneventful, and the patient was discharged on postoperative day 11. This case highlights diagnostic and therapeutic strategies for this rare entity and offers clinical insights for similar presentations.

## Background

Right ventricular myxoma (RVM) is exceptionally rare, accounting for approximately 3%–4% of all cardiac myxomas (1, 2). Its specific location within the right ventricle can cause hemodynamic disturbances and complications, including right heart failure. Tumor fragments or surface thrombi may embolize into the pulmonary circulation, leading to pulmonary embolism and potentially fatal outcomes. Surgical resection remains the primary treatment, with generally favorable prognoses. This report describes a patient diagnosed with RVM complicated by pulmonary embolism but without clinical or biochemical evidence of Cushing's syndrome, aiming to contribute to clinical understanding and management.

## Case description

**Figure 1:**
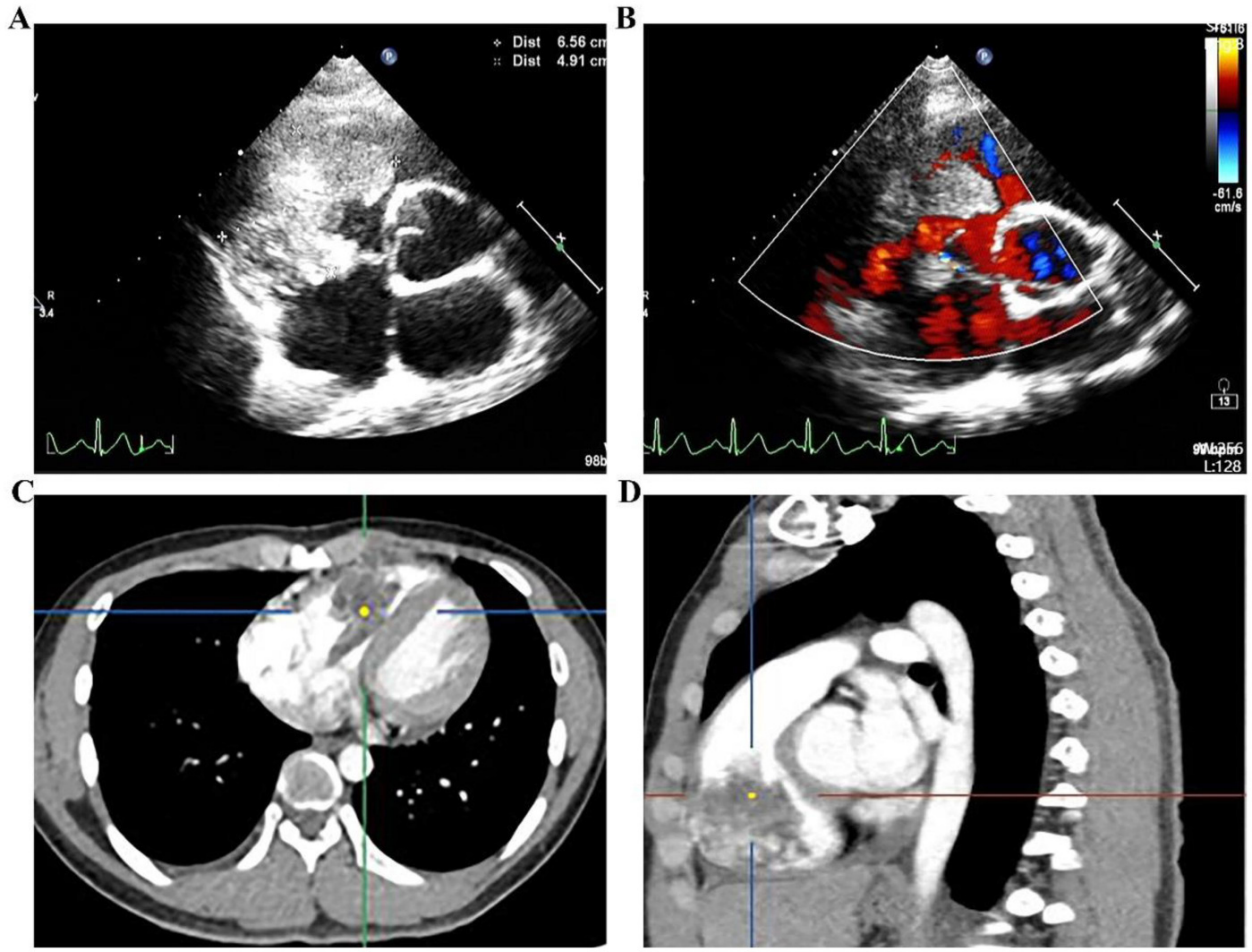
Echocardiography and enhanced chest CT demonstrating a right ventricular space-occupying lesion. **(A,B)** Echocardiogram; **(C,D)** enhanced chest CT.

An 18-year-old male presented with sudden-onset chest tightness and a mild non-productive cough during a basketball game one week prior. He reported no fever or other discomfort. On May 23, 2022, transthoracic echocardiography revealed an abnormal right ventricular mass measuring 7 cm × 6.5 cm × 4.9 cm, which was strongly suggestive of myxoma, accompanied by mild tricuspid regurgitation (Figures 1A,B). The patient had no history of chronic illness, infectious disease, or allergies.

### Physical examination

Vital signs were stable (body temperature, 36.7 °C; pulse, 87/min; respiratory rate, 18/min; blood pressure, 116/73 mmHg). Height was 175 cm and weight 52 kg. No stigmata of Cushing's syndrome (moon face, ruddy complexion, centripetal obesity, or dorsocervical fat pad) were observed. Jaundice, cyanosis, lymphadenopathy, or peripheral edema was not present. The chest was symmetrical, breath sounds were clear bilaterally, and the cardiac rhythm was regular without thrills or abnormal precordial findings. The abdominal examination was unremarkable, and the patient was fully ambulatory.

**Figure 2:**
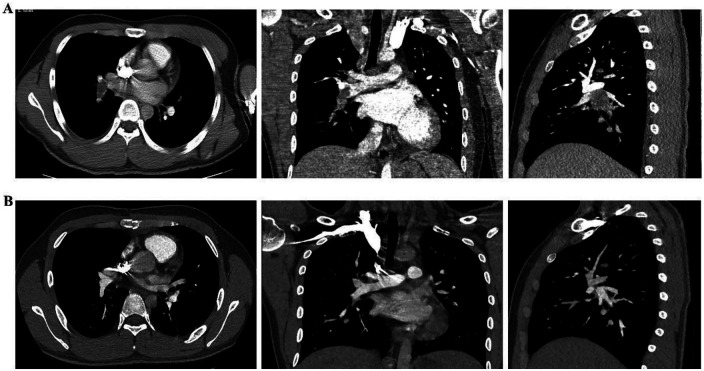
Pulmonary artery CTA showing the preoperative and postoperative conditions of pulmonary artery thrombosis. **(A)** Preoperative pulmonary artery CTA; **(B)** postoperative pulmonary artery CTA.

After admission, on May 25, 2022, contrast-enhanced chest computed tomography (CT) revealed a patchy, heterogeneously enhancing lesion in the right ventricle, measuring approximately 4.7 cm × 4.3 cm, closely adherent to the ventricular wall and interventricular septum. A nodular filling defect (approximately 2.3 cm × 1.6 cm) was identified in the right lower pulmonary artery trunk, with distal branches markedly attenuated and sparse (Figures 1C,D). Pulmonary artery CTA confirmed the absence of main pulmonary artery dilation (inner diameter ∼2.66 cm), filling defect in the right lower pulmonary artery (2.3 cm × 1.6 cm), and attenuation of distal branches (Figure 2). Endocrine evaluation showed that adrenocorticotropic hormone (ACTH), cortisol rhythm, and urinary free cortisol levels were within normal limits. Abdominal ultrasonography revealed no abnormalities.

### Surgical procedure

**Figure 3:**
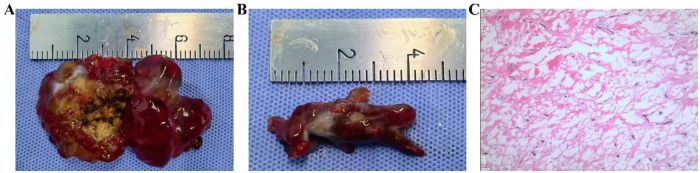
Intraoperative findings and postoperative pathological examination. **(A)** Ventricular myxoma; **(B)** pulmonary artery tumor thrombus; **(C)** histopathological confirmation of myxoma.

On May 27, 2022, the patient underwent cardiopulmonary bypass surgery, which included complete resection of the cardiac tumor, pulmonary arteriotomy with thrombectomy, tricuspid valvuloplasty, and definitive cardiac repair. Under general anesthesia, with the patient supine and following routine sterilization and draping, a midline sternotomy was performed. After systemic heparinization, the sternum was divided longitudinally. The pericardium was incised, exposing the heart, which demonstrated right-sided enlargement without evidence of aortic root thrill. Cannulation was achieved via the superior and inferior venae cava and the ascending aorta. Cardiopulmonary bypass was initiated, and systemic cooling to 32 °C was performed. After aortic cross-clamping, cold cardioplegic solution was administered via the aortic root to induce cardiac arrest. A right atriotomy was then performed, with satisfactory coronary sinus return during cardioplegia. The interatrial septum was incised to facilitate left-heart venting. The tricuspid annulus was dilated, and a large friable myxomatous tumor was observed protruding from the right ventricle into the right atrium through the tricuspid orifice. The tumor filled most of the right ventricular cavity. The mass was partially excised piecemeal, and the pedicle, originating from the right ventricular free wall, was completely resected at its base (Figures 3A,B). The right heart chambers were thoroughly irrigated. A 2.5 cm × 0.3 cm autologous pericardial strip was fashioned into an annuloplasty ring, and tricuspid valvuloplasty was performed. A longitudinal incision was made in the right pulmonary artery trunk, revealing an embolus measuring approximately 2 cm × 1.5 cm, which was completely removed under suction. After repeated irrigation, the pulmonary arteriotomy was closed with a 5.0 Prolene suture. Cardiopulmonary bypass was discontinued, and the chest was closed in layers following meticulous hemostasis.

### Postoperative course

**Figure 4:**
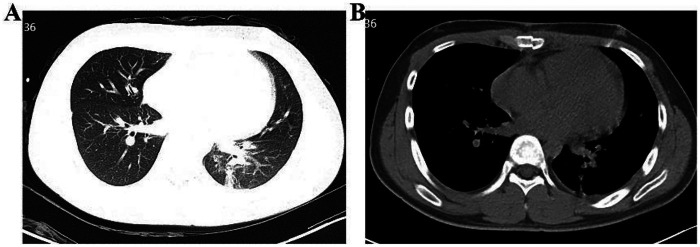
Postoperative non-contrast chest CT demonstrating lung re-expansion without evidence of infection. **(A)** Lung window; **(B)** Mediastinal Window.

**Figure 5:**
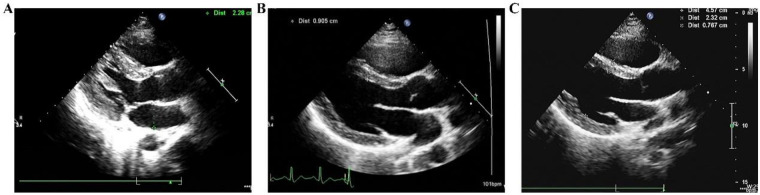
Serial echocardiograms at different periods showing no abnormal intracardiac masses. **(A)** August 31, 2022; **(B)** November 22, 2022; January 10, 2023.

The patient received prophylactic antibiotics (cefaloridine), acid suppression therapy, nebulization, expectorants, antitussives, analgesics, and anticoagulation. Histopathological examination confirmed the presence of a right ventricular myxoma and a pulmonary artery myxomatous embolus (Figure 3C). On June 6, 2022, non-contrast chest CT demonstrated postoperative changes, mild bilateral pleural effusion, and lower lobe inflammation (Figure 4). Pulmonary CTA performed on June 8, 2022 showed no residual thrombus in the right lower pulmonary artery (Figure 2). Subsequent echocardiograms on August 31, November 22, 2022, and January 10, 2023, consistently demonstrated no residual right ventricular mass, preserved systolic function (Figure 5), and intact tricuspid valvuloplasty.

## Discussion

Left atrial myxoma is the most common subtype, whereas RVM is exceedingly rare. Several cases of RVM complicated by pulmonary tumor embolism and associated with Cushing's syndrome have been reported, with favorable postoperative outcomes (1, 2, 4). In contrast, our patient was young and did not fall within the typical age range for myxoma. This case describes a giant RVM with a pulmonary artery tumor embolism but without Cushing's syndrome. To the best of our knowledge, no prior reports have documented a successful single-stage procedure combining tumor resection, pulmonary thrombectomy, and tricuspid valve repair for this condition. In our patient, timely echocardiographic evaluation was essential for the early detection of right-sided cardiac tumors, particularly in the absence of overt endocrine symptoms.

The success of this case hinged on the surgical team's proactive and comprehensive single-stage approach. Under cardiopulmonary bypass, via a right atrial incision, the team achieved complete resection of the giant ventricular tumor together with its pedicle, performed a longitudinal pulmonary arteriotomy to extract distal emboli, and concurrently repaired the tricuspid valve. This integrated strategy offered considerable benefits by simultaneously addressing three critical issues: the tumor mass, embolic risk, and secondary valve dysfunction (resulting from tricuspid annular dilation due to the tumor's size). Consequently, postoperative complications and the need for reoperation related to residual embolism or valvular regurgitation were minimized. Meticulous intraoperative irrigation of the cardiac chambers and pulmonary artery was also vital to prevent tumor seeding or retention of embolic fragments.

Pathological analysis definitively established the diagnosis of cardiac myxoma and myxomatous emboli, serving as the diagnostic gold standard. The patient demonstrated favorable short- and mid-term outcomes. Postoperative echocardiography confirmed the complete removal of the right ventricular mass, and pulmonary CTA verified the resolution of the embolism, with only mild tricuspid regurgitation persisting. The left ventricular ejection fraction remained within the normal range and showed an improving trend. These findings indicate that even in complex presentations involving a large tumor with an embolism, meticulous and radical surgical intervention can achieve near-complete anatomical and functional recovery, enabling the patient to resume normal life and attesting to the curative potential of surgery.

Nevertheless, this case has limitations. The exclusion of Cushing's syndrome was based solely on clinical symptoms and initial laboratory findings, without a comprehensive endocrine assessment. In addition, the follow-up duration was relatively short. In summary, this case offers valuable clinical guidance for managing the rare entity of RVM with pulmonary artery embolism. It reaffirms that early diagnosis and an aggressive, definitive single-stage surgical procedure are crucial for achieving a cure. Preoperative workup should include comprehensive imaging such as echocardiography and CTA to delineate tumor extent and embolic burden. Intraoperatively, complete excision of the tumor and emboli should be pursued, along with appropriate management of concomitant valvular pathology. Long-term regular imaging surveillance is necessary postoperatively to monitor recurrence. This successful outcome highlights the central role of a multidisciplinary approach anchored in cardiac surgery for the management of complex cardiac tumors.

## Data Availability

The original contributions presented in the study are included in the article/Supplementary Material, further inquiries can be directed to the corresponding authors.
